# Planning TTFields treatment using the NovoTAL system-clinical case series beyond the use of MRI contrast enhancement

**DOI:** 10.1186/s12885-016-2890-0

**Published:** 2016-11-04

**Authors:** Jennifer Connelly, Adília Hormigo, Nimish Mohilie, Jethro Hu, Aafia Chaudhry, Nicholas Blondin

**Affiliations:** 1 Froedtert Hospital and the Medical College of Wisconsin, Milwaukee, WI USA; 2 Department of Neurology, Medicine and Neurosurgery, The Icahn School of Medicine at Mount Sinai and The Tisch Cancer Institute, New York, NY USA; 3 University of Rochester Medical Center, Rochester, NY USA; 4 Cedars-Sinai Medical Center, Los Angeles, CA USA; 5 Novocure Global Medical Affairs, New York, NY USA; 6 Associated Neurologists of Southern Connecticut, Fairfield, CT USA

**Keywords:** Glioblastoma, NovoTAL, NovoTTF-100A System, Treatment planning, TTFields, Tumor treating fields

## Abstract

**Background:**

Gliomas are the most common primary brain tumors in adults and invariably carry a poor prognosis. Recent clinical studies have demonstrated the safety and compelling survival benefit when tumor treating fields (TTFields) are added to temozolomide for patients with newly diagnosed glioblastoma. TTFields are low-intensity, intermediate frequency (200 kHz) alternating electric fields, delivered directly to a patient’s brain through the local application of non-invasive transducer arrays. Experimental simulations have demonstrated that TTFields distribute in a non-uniform manner within the brain. To ensure patients receive the maximal therapeutic level of TTFields at the site of their tumor, tumor burden is mapped and an optimal array layout is personalized using the NovoTAL software. The NovoTAL software utilizes magnetic resonance imaging (MRI) measurements for head size and tumor location obtained from axial and coronal T1 postcontrast sequences to determine the optimal paired transducer array configuration that will deliver the maximal field intensity at the site of the tumor. In clinical practice, physicians planning treatment with TTFields may determine that disease activity is more accurately represented in noncontrast-enhancing sequences. Here we present and discuss a series of 8 cases where a treating physician has utilized non-contrast enhancement and advanced imaging to inform TTFields treatment planning based on a clinical evaluation of where a patient is believed to have active tumor. This case series is, to our knowledge, the first report of this kind in the literature.

**Case presentations:**

All patients presented with gliomas (grades 2–4) and ranged in age from 49 to 65 years; 5 were male and 3, female. Each patient had previously received standard therapy including surgery, radiation therapy and/or chemotherapy prior to initiation of TTFields. The majority had progressed on prior therapy. A standard pre- and postcontrast MRI scan was acquired and used for TTFields treatment planning.

**Conclusion:**

This paper details important approaches for integrating clinical considerations, nonmeasurable disease and advanced imaging into the treatment planning workflow for TTFields. As TTFields become integrated into standard care pathways for glioblastoma, this case series demonstrates that treatment planning beyond the extent of contrast enhancement is clinically feasible and should be prospectively compared to standard treatment planning in a clinical trial setting, in order to determine the impact on patient outcomes.

## Background

Despite significant advances in the detection, imaging and treatment planning for brain tumors, overall prognosis remains bleak and there remain no curative treatments for high-grade gliomas. There is a significant unmet need for life-extending therapies that can preserve a patient’s quality of life, given that patients with glioblastoma multiforme (GBM) have a median survival of approximately 16 months, even with aggressive therapy [1–3]. Tumor treating fields (TTFields) are low-intensity (1–3 V/cm), intermediate frequency (200 kHz), alternating electric fields approved for the management of patients with both newly diagnosed and recurrent GBM [4, 5]. Optune™ (NovoTTF-100A System) is a Food and Drug Administration (FDA) approved device that locally delivers TTFields to a patient’s head through paired transducer arrays, which are worn continuously on the scalp. At therapeutic field intensities, TTFields will selectively target dividing cancer cells, sparing quiescent cells [6, 7].

TTFields fall within the spectrum of nonionizing radiation and differ in their biologic behavior compared with radiation therapy (RT). In general, high energy gamma radiation has a frequency in the order of exahertz (10^20^), with a wavelength of picometers (10^−19^), less than the diameter of an atom [8]. As the frequency of TTFields is much lower at 200 kHz, and the wavelength much longer (~1 mile), TTFields cannot be precisely focused and delivered to discrete regions of the brain in the same focal manner as RT [9, 10]. Treatment can, however, be optimized to ensure that field intensity is maximal at the site of a tumor [11–13], a process which involves planning with the NovoTAL System software.

Conventional treatment planning for TTFields, which is a requisite for all patients commencing therapy, is performed using axial and coronal T1-post contrast MRI measurements and the NovoTAL™ system. The methods for performing treatment planning have been described previously [11], but in brief, consist of obtaining 20 measurements delineated on T1 postcontrast sequences from the patient’s most recent MRI scan. Coordinate dimensions are obtained for head size using T1 postcontrast sequences, on a slice at the superior level of the orbit. A box is drawn at the level of the scalp. Head size measurements are obtained for the maximal anterior-posterior (A-P), right to left lateral (R-L) and right to anatomic midline distances and are recorded on a planning worksheet. The size of the cerebrum is estimated from a coronal T1 postcontrast slice at the level of the external auditory canal, commencing from a reference frame drawn at the level of the scalp. The horizontal margin of this box extends inferiorly to the lower margins of temporal lobe. Superior-to-inferior, R-L and right to anatomic midline measurements on coronal views are obtained and recorded on the worksheet. In conventional treatment planning, tumor location coordinates from similar reference frames are obtained in both axial and coronal views, selecting the T1 postcontrast slices that contain the maximal extent of enhancing disease. Reference frames are drawn around the head at the level of the scalp, and the same 3 dimensions as described for head size are obtained in the axial view and coronal views. In addition in the axial sequence, distances to the edge of tumor are obtained from the right frame to near and far tumor margins, and from the anterior frame to the near and far tumor margins. In the coronal slice, tumor coordinates are obtained from the right frame to the near and far tumor margins, and from the top of the frame to the near and far tumor margins. All measurements are rounded to the nearest mm and recorded on the planning worksheet. Once complete, the measurements are entered into the NovoTAL software in order to generate a personalized transducer array layout map. The layout map is used to direct TTFields treatment.

As mentioned, conventional treatment mapping is typically targeted to the margins of a lesion as represented by an area of maximal contrast enhancement viewed on axial and coronal MRI frames. However, the contrast enhancement defines areas where the blood–brain barrier is disrupted and mapping treatment exclusively on postcontrast imaging does not take into account nonmeasurable disease that is nonenhancing and which may extend beyond the margins of these highly infiltrative tumors. Therefore, in clinical practice, constraining planning to the extent of enhancing disease may not target all the clinically relevant disease foci that can be determined with other sequences such T2 FLAIR and DWI. Response assessment in high grade gliomas is complex due to tumor heterogeneity and the impact of prior therapies on imaging. The Response Assessment in Neuro-Oncology (RANO) are the current criteria used by the neurooncology community in the clinical practice and clinical trial settings, and these criteria have incorporated the identification of nonenhancing, infiltrative tumor into response assessment to address the limitations of using only contrast enhancing images [14]. Consequently, it is appropriate for treating physicians to incorporate their knowledge of both enhancing and nonenhancing disease into the treatment planning for patients receiving TTFields. Given the paucity of literature on treatment planning approaches with TTFields, we report a series of cases demonstrating feasibility of using alternate MRI sequences when planning therapy with TTFields.

## Case presentation

### Practice scenario 1: selective use of contrast-enhancement to map to areas likely to represent active disease

#### Case A

**Fig. 1:**
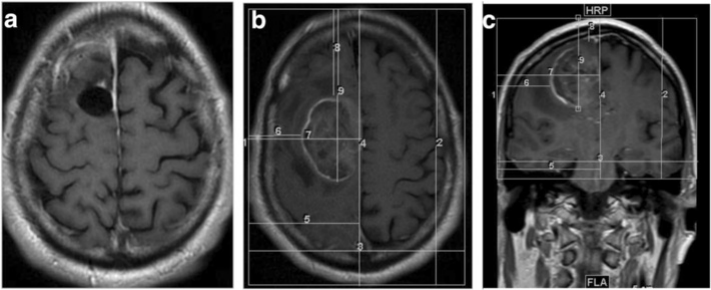
**a**, **b & c**: Figure **a** demonstrates post-operative loss of contrast-enhancing disease in a glioblastoma patient post gross total resection. **b** and **c** show pre-operative axial and coronal views of the tumor. NovoTAL mapping was performed using pre-operative T1 postcontrast sequences in a standard manner. Head size measurements were determined on axial and coronal views (not shown). In figure **b**, axial tumor location was determined measuring from a reference frame drawn at the level of the scalp. Axial tumor locations measurements are shown A-P (*2*), R-L (*3*), R-midline (*5*), right to near tumor margin (*6*), right to far tumor margin (*4*), front to near tumor margin (*8*), front to far tumor margin (*9*). Figure **c** shows tumor location measurements obtained in coronal view. All measurements originate from a reference frame drawn at the level of scalp, extending inferiorly to the lower margins of temporal lobe. The coronal tumor location measurements consist of a superior to inferior measurement (*2*), R-L (*3*), right to midline (*5*), right to close (*6*) and right to far (*7*) tumor margins, and superior to close (*8*) and superior to far (*9*) tumor margin measures

A 54-year-old man with a longstanding history of seizures developed new left-sided weakness and was found to have a brain tumor. He underwent a gross total resection and pathology confirmed a diagnosis of GBM with both astrocytic and oligodendroglial features, MGMT unmethylated, IDH1 wild-type and no loss of 1p19q. He was treated with conventional chemo-RT and opted to add TTFields to his maintenance temozolomide. His postradiation MRI showed no clear contrast enhancement and only minor fluid-attenuated inversion recovery (FLAIR) changes. MRI measurements for NovoTAL treatment planning were thus determined using the preoperative scans (Fig. 1a-c).

#### Case B

**Fig. 2:**
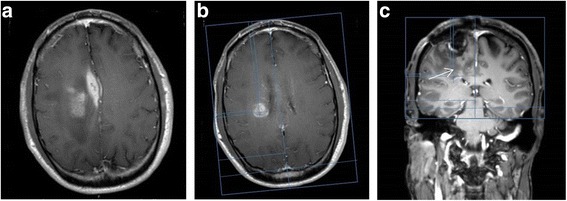
**a**, **b & c**: Figure **a** demonstrates parenchymal enhancement in a Gliadel-treated region (shown by the *arrow*), and an adjacent subdural collection along the right falx in the T1 postcontrast sequence. Figure **b** demonstrates an enhancing nodule in the right periventricular region which correlated to new neurological symptoms. Axial tumor location measurements were performed targeting the TTFields to this specific lesion. Standard measurements were performed from a reference frame drawn at the outer margin of the scalp. Figure **c** represents the coronal slice selected for TTFields treatment planning. A reference frame is drawn at the level of the scalp, extending inferiorly to the lower margin of frontal lobe. At the clinician’s discretion, tumor location coordinates are determined for the area thought to represent active tumor (*arrow*)

A 49-year-old man developed left arm weakness. Imaging revealed a ring-enhancing nodule in the posterior right frontal lobe. He underwent a gross total resection with pathology confirming a GBM, MGMT unmethylated. He was treated with conventional chemo-RT, followed by 1 cycle of maintenance temozolomide. The patient experienced disease progression and was started on bevacizumab, with continuation of temozolomide. After completing 6 cycles of temozolomide, he had a further progression and underwent a repeat tumor resection, with placement of Gliadel wafers. Following the surgery, he received bevacizumab in combination with dose-intense (daily) temozolomide for 2 months. Subsequently, the patient developed new symptoms of worsening left-sided weakness. Imaging performed at that time demonstrated three areas of abnormal enhancement, including the resection cavity with Gliadel wafers, an enhancing area in the medial right frontal lobe suggestive of a subdural collection, and a new enhancing mass in the deep right hemisphere (Fig. 2). NovoTAL mapping was performed utilizing measurements of the deep tumor, and TTFields therapy was initiated.

#### Clinical commentary

Conventional NovoTAL treatment planning is focused on determining coordinates from a fixed frame to the proximal and distal borders of contrast enhancement viewed on axial and coronal MRI images. In patients newly diagnosed with glioblastoma, TTFields are commenced in combination with maintenance temozolomide and treatment is typically planned using a 4-week post-RT MRI. In Case A, the post-RT MRI did not reveal any abnormal contrast enhancement. Therefore, the treating physician utilized the preoperative postcontrast scan to perform NovoTAL mapping and generate an array layout map to maximize electrical field intensity in the region of preoperatively visible tumor. In Case B, three areas of abnormal enhancement were visualized on the TTFields planning MRI. Based on the patient’s treatment history and new clinical symptoms, the treating physician determined that the new nodule in the deep right hemisphere represented the area of active disease. The enhancement in the resection cavity with Gliadel wafers and the subdural collection were deemed to represent treatment effect without active disease. Therefore, NovoTAL mapping was performed on the region of contrast enhancement believed to be most reflective of active disease. In both cases, the treating physician made a determination that T1-postcontrast enhancement either underestimated or overestimated extent of disease and performed treatment mapping based on where they understood most active disease was present.

### Practice scenario 2: mapping to active disease represented by T2/FLAIR sequences

#### Case C

**Fig. 3:**
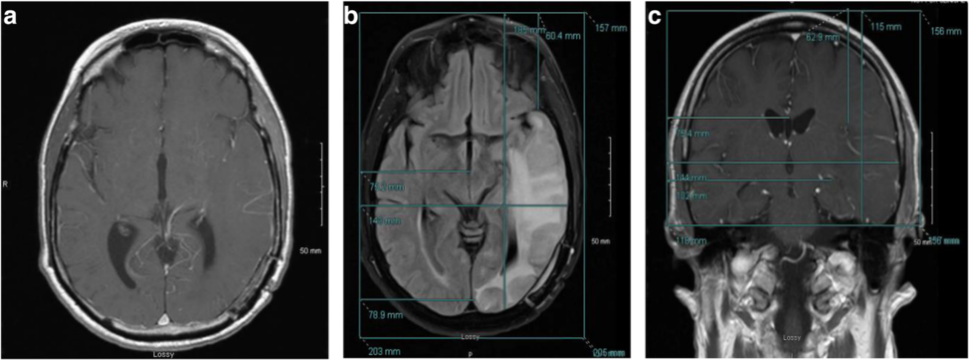
**a**, **b & c**: Figure **a** demonstrates lack of enhancing disease on an axial T1 postcontrast scan in a glioblastoma patient experiencing disease progression following 5 months of treatment with bevacizumab. Figures **b** shows the corresponding slice on the axial FLAIR sequence demonstrating significant FLAIR signal abnormality. As such, TTFields planning was performed on the FLAIR sequence. Measurements were obtained from a reference frame drawn at the level of the scalp in a standard manner, and measuring from the right frame to the near and far borders of the FLAIR signal abnormality. Figure **c** depicts mapping performed in the coronal plane to a corresponding area of hypodensity on the T1 postcontrast sequence. All measurements are obtained from a reference frame drawn at the level of the scalp, extending inferiorly to the lower margin of temporal lobe

A 65-year-old man initially presented with word processing difficulties. MRI of the brain revealed a heterogeneously enhancing left temporo-occipital mass. He underwent an awake craniotomy with subtotal resection. Pathology was consistent with GBM, IDH1 wild-type, MGMT unmethylated. He was treated with conventional chemo-RT. His postradiation MRI showed disease progression and bevacizumab was initiated, resulting in a radiographic improvement. After 5 months of treatment, his MRI demonstrated progression of the nonenhancing tumor and the patient declined clinically. At progression, he was started on dexamethasone, temozolomide, and TTFields. NovoTAL mapping was performed targeting the new nonenhancing FLAIR abnormality (Fig. 3).

#### Case D

**Fig. 4:**
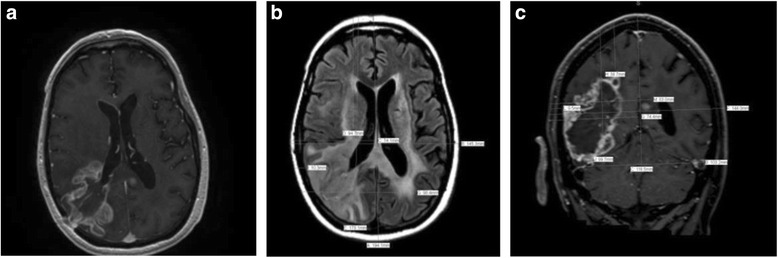
**a**, **b & c** demonstrate treatment planning for a recurrent glioblastoma patient with diffuse infiltrating disease. Figure **a** shows the T1 with contrast axial slice demonstrating enhancing tumor. Figure **b** demonstrates more diffuse FLAIR signal abnormality, including within the contralateral lobe. As such, treatment is planned using the FLAIR sequence. Standard measurements were performed from a reference frame drawn at the outer margin of the scalp. Tumor location coordinates are obtained on the axial slice measuring from the right frame to the proximal and distal extents of FLAIR abnormality. In the absence of a coronal FLAIR sequence, coronal treatment planning is performed using the T1 postcontrast sequence (figure **c**). In this slice, the inferior margin of the reference frame is drawn to the lowest visible level of tentorium

A 55-year-old man developed progressively worsening headaches, nausea, and vomiting. Imaging revealed a large right temporal-parietal mass. A biopsy was performed, confirming a GBM, IDH wild-type, MGMT unmethylated. After 4 cycles of temozolomide, he developed a visual field deficit, more disorientation, right–left confusion and difficulty with memory. He was treated with conventional chemo-RT followed by 4 cycles of adjuvant temozolomide. An MRI then revealed new local growth of mass, as well as the development of a small new enhancing lesion in the splenium of his corpus callosum. There was significant T2 hyperintensity in this area consistent with infiltrative disease. Due to this significant nonenhancing disease, NovoTAL mapping was performed incorporating the region of T2 signal change (Fig. 4).

#### Case E

**Fig. 5:**
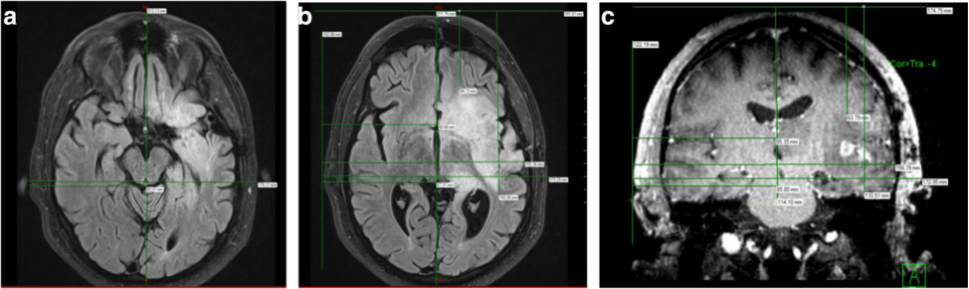
**a**, **b & c** show a largely noncontrast enhancing tumor. Head size measurements are performed on the FLAIR sequence. The axial coordinates for head size are shown in figure **a**. NovoTAL mapping was performed exclusively using figure **b** axial and figure **c** coronal FLAIR images. In the axial view figure **b**, a reference frame is drawn on the slice demonstrating the greatest extent of FLAIR abnormality. Tumor coordinates are obtained from the right frame to the near and far edges of FLAIR abnormality at the discretion of the treating physician. In figure **c**, coronal tumor location coordinates are obtained from a reference frame drawn at the level of the scalp which extends inferiorly to the lowest margin of temporal lobe. Distances to the tumor margin are drawn at the discretion of the treating physician to include all the region that is thought to contain active tumor

A 51-year-old man developed headaches and subsequently had a seizure. Imaging revealed an area of abnormal T2 hyperintensity in the left insular region. He underwent a biopsy, which was nondiagnostic. Four months later, repeat imaging demonstrated an interval expansion of abnormal T2 hyperintensity. A second biopsy was performed, confirming an anaplastic astrocytoma, IDH1 wild-type. He was treated with conventional chemo-RT followed by 7 cycles of adjuvant temozolomide. Imaging subsequently revealed a new area of abnormal enhancement in the medial left temporal lobe. He elected to initiate treatment with dose-intense temozolomide and concurrent TTFields therapy. NovoTAL mapping was performed utilizing the extent of the T2 hyperintense signal, rather than focusing solely on the small area of abnormal enhancement (Fig. 5).

#### Case F

**Fig. 6:**
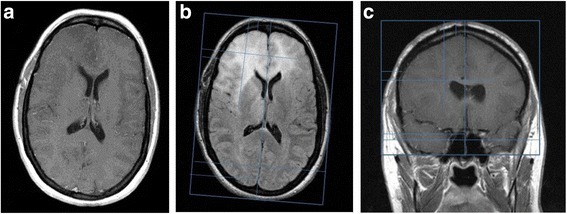
**a**, **b & c** demonstrate treatment planning in a patient with exclusively nonmeasurable disease: Figure **a** is the axial T1 post-contrast slice demonstrating no contrast enhancement. Figure **b** is the corresponding axial FLAIR sequence was used to map the tumor coordinates. A reference frame is drawn around the level of the scalp and tumor location coordinates are obtained from the right framebased on the extent of abnormal T2 signal. Figure **c** depicts the coronal treatment planning utilizing the T1 post-contrast sequence. The area of FLAIR signal abnormality corresponds with an area of T1 hypointensity on the coronal sequence. Treatment is planned to the boundaries of the T1 hypointensity at the treating physician’s discretion. Tumor location coordinates are obtained in a standard fashion from a reference drawn at the level of the scalp. The reference frame encompasses all of the supratentorial brain so the lower boundary is extended to the inferior margin of temporal lobe in this slice

A 49-year-old woman developed worsening headaches, dizziness, and falls. Imaging revealed an area of diffuse abnormal T2 hyperintense signal centered in the right frontal lobe, extending into the left frontal lobe and deep right hemisphere. She underwent a stereotactic biopsy of the right frontal lobe. Pathology revealed an astrocytoma grade II, IDH-1 mutated. She opted for treatment with conventional temozolomide cycles, and concurrent TTFields therapy. NovoTAL mapping was performed utilizing the entire area of abnormal T2 signal in the right frontal lobe as the mass lesion (Fig. 6).

#### Clinical commentary

Malignant gliomas are often composed of a heterogeneously enhancing mass and an infiltrative nonenhancing FLAIR abnormality. This FLAIR abnormality needs to be distinguished from peritumoral edema and/or treatment effect. According to the RANO Criteria, FLAIR changes are incorporated in the tumor measurements with the recognition that these often represent nonenhancing infiltrative and progressive tumor [14]. It is at the discretion of the treating physician to determine which areas most likely represent infiltrative tumor and should be the target of the TTFields.

Case C represents a common radiographic pattern seen in glioblastoma bevacizumab failure, characterized by lack of contrast enhancement and progression of abnormal T2 signal or FLAIR. The treating physician used the new area of FLAIR as the target of the TTFields. Case D represents a scenario where the treating physician used abnormal T2 signal on the FLAIR series to delineate the tumor boundary. As the bulk of the tumor did not enhance with contrast, abnormal FLAIR hyperintensity was used to delineate the tumor boundary.

The natural history of grade II and III gliomas is such that, overtime (months to years), they can often transform to a malignant phenotype. The management of anaplastic astrocytomas remains controversial, but most clinicians opt to treat patients with the same regimen used to treat glioblastoma; adjuvant chemo radiation followed by maintenance temozolomide. Although TTFields have not been prospectively tested in randomized controlled trials in low grade gliomas and are not FDA approved in this setting, they have been used off-label to treat these patients when other options have been exhausted, given the highly limited treatment options available for this population. Radiographically, low-grade gliomas tend to be nonenhancing and manifest with abnormal T2 signal or FLAIR. In these scenarios, as in Case E and F, axial and coronal FLAIR imaging was used for mapping. If coronal T2 or FLAIR are unavailable, the coronal T1-weighted imaging can be used, in which case the tumor often appears hypodense. In Case E, a diffuse astrocytoma, there was no contrast enhancement seen on MRI. Axial FLAIR images, demonstrating T2 hyperintensity in affected areas of the brain, were used to obtain mapping coordinates. No coronal T2 or FLAIR sequence was available, so the coronal T1-weighted image was used to estimate the central area of the tumor. There was some evidence of T1 hypointensity, corresponding to T2 hyperintensity.

In Case F, there was no abnormal contrast enhancement seen on MRI, so the axial T2- and T1-post, as well as the coronal T1-post were used for NovoTAL mapping. The mass was T1 hypointense and T2 hyperintense to normal brain, allowing for drawing of the coordinates up to the signal change. The cases outlined above demonstrate how treating physicians can appropriately incorporate nonenhancing disease into TTFields treatment plans.

### Practice scenario 3: incorporating perfusion/diffusion imaging in treatment planning

#### Case G

**Fig. 7:**
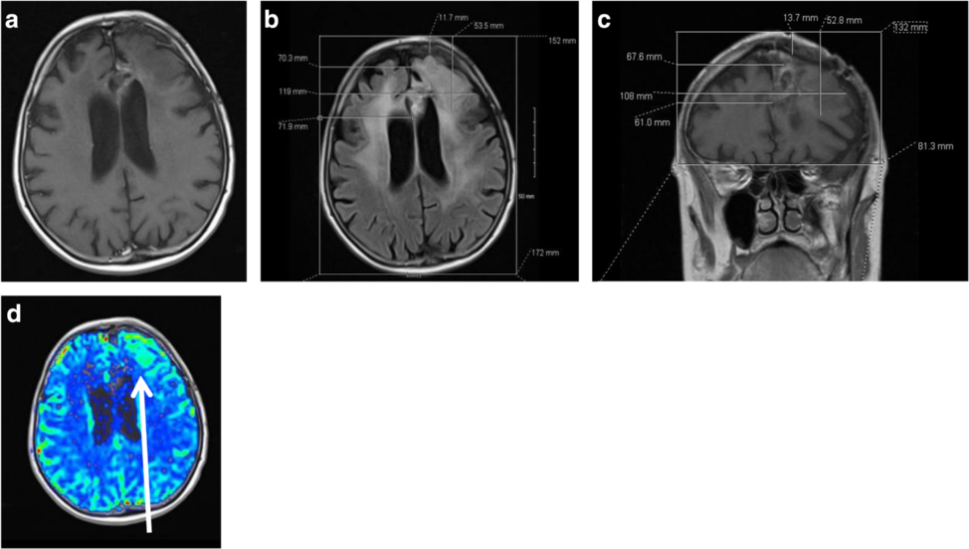
**a**, **b**, **c & d**. Figures **a**-**d** depict treatment planning in a recurrent glioblastoma patient presenting with an area of increased perfusion on sequential imaging. Figure **a** shows lack of enhancement on the axial T1 postcontrast sequence. Figure **b** shows axial treatment planning performed on the corresponding slice in the FLAIR sequence, where diffuse signal abnormality is present. Measurements are obtained from a reference frame drawn at the level of the scalp. Tumor coordinates are determined in a standard manner correlating the region of FLAIR signal abnormality with the area of increased perfusion observed on MR perfusion (figure **d**). Figure **c** shows the corresponding area of hypodensity on the coronal T1 with contrast sequence which is used for treatment planning in the absence of a coronal FLAIR sequence. All measurements are obtained from a reference frame drawn at the level of scalp, extending inferiorly to the lowest margin of frontal lobe. In anterior coronal sections, the reference frame should encompass all of the cerebrum at this level. Tumor location measurements are obtained in a standard fashion extending from the right frame to the edges of the T1 hypo intensity. Figure **d** shows an area of increased perfusion (*white arrow*) in the left frontal lobe on MR perfusion indicative of tumor recurrence

A 65-year-old woman developed progressively worsening cognitive and speech impairments. Imaging revealed a large, heterogeneously enhancing left frontal mass extending to the corpus callosum. She underwent a subtotal resection, with pathology confirming a GBM, MGMT methylated, and EGFRvIII positive. She was treated with conventional chemo-RT and adjuvant temozolomide along with an investigational agent through a clinical trial. After her second recurrence while on bevacizumab, MRI demonstrated an enlarging abnormal T2 hyperintense area. This region also had significant increase in blood flow on MR perfusion (Fig. 5), suggesting active tumor growth. She opted for continuation of bevacizumab and the addition of lomustine and TTFields. NovoTAL mapping was performed utilizing the new nonenhancing T2 signal abnormality that had developed with hyperperfusion. A coronal FLAIR image was unavailable, so an area of hypodensity on coronal T1 with contrast was also used in mapping (Fig. 7).

#### Case H

**Fig. 8:**
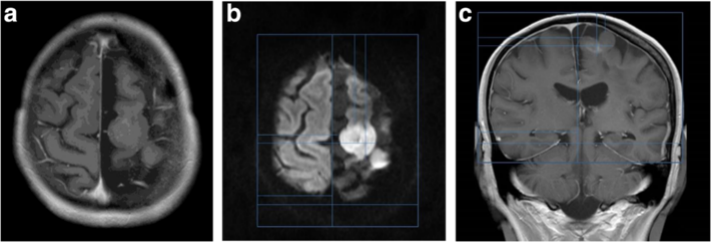
**a**, **b & c** depict treatment planning in a patient with recurrent anaplastic astrocytoma incorporating DWI sequences. Figure **a** shows an axial T1 postcontrast slice demonstrating punctate enhancement. Head size measurements were performed in a standard manner on T1 sequences (not shown). Figure **b** shows the axial DWI sequence with a clear area of hyperintense signal in the region of active disease which was used to obtain axial tumor location measurements. A reference frame is approximated around the head at the level of the scalp. Tumor location coordinates are measured from the right frame to the edges of the DWI signal abnormality thought to represent active tumor at the discretion of the treating physician. Figure 8c shows the coronal T1 postcontrast slice used to map tumor coordinates in the coronal plane. Treatment was planned measuring from a reference frame drawn at the level of the scalp extending to the lowest margin of visible tentorium. Tumor location coordinates are measured from the right frame to the edges of faint enhancement and gyral thickening

A 49-year-old woman developed progressively worsening speech impairment. Imaging revealed a nodular area of DWI-hyperintense signal (restricted diffusion) in the deep left frontal lobe. She underwent a stereotactic biopsy confirming an anaplastic astrocytoma, IDH1 wild-type, and MGMT unmethlyated. She was treated with conventional chemo-RT. During the fourth week of radiation, she experienced worsening neurological symptoms, complete expressive aphasia, and right-sided hemiplegia. She was started on concurrent bevacizumab and was able to complete her course of chemo-RT thereafter continuing on bevacizumab. After 11 months of therapy, she developed new speech impairment. MRI demonstrated a new nodule of hyperintense DWI signal in the high-left frontal lobe, consistent with disease progression. It was recommended that TTFields be added as adjunctive therapy. NovoTAL mapping was performed utilizing the DWI sequence, which was correlated to the axial and coronal T1-postcontrast series (Fig. 8).

#### Clinical commentary

In some cases, aggressive tumor growth can be better visualized on MR-perfusion and DWI sequences. MR-perfusion represents increased blood flow to actively growing tumor, whereas DWI-hyperintensity represents a hypercellular environment. This is particularly useful in the era of antiangiogenic therapies, in which patients often have little or no contrast enhancement on MRI at the time of tumor progression. Utilization of these advanced MR modalities can guide the target of the TTFields to the area of most active tumor. In Case G, the area of increased activity on MR-perfusion narrowed the region of interest on FLAIR. In Case H, the hyperintense DWI signal, which correlated with the patient’s symptoms, defined the target on T1-postcontrast. In both cases, the treating physician used advanced MRI techniques to help target the TTFields.

## Discussion

The NovoTAL System is an algorithmic software program that creates optimal transducer array layouts based on patient head size and tumor location measurements obtained from an MRI [11]. Electric field distribution within the brain is nonuniform, and is a function of the direction of a field, individual dielectric properties of adjacent tissue structures, and the orientation of the interfaces between them [9, 10]. The algorithm will compute the optimal paired configuration of transducer arrays that should be applied directly to a patient’s shaved scalp that will locally deliver the highest intensity of TTFields at the site of a tumor. The NovoTAL System was FDA approved in 2013 on the basis of a user study that included a total of 14 neuro-oncologists, neurosurgeons, and medical oncologists who were trained on treatment planning and then had to independently generate treatment maps for 5 blinded GBM cases [11]. The concordance between physician mapping and gold-standard in-house mapping performed by the manufacturer’s clinical team was excellent, with an R^2^-correlation coefficient for 20 individual measuring parameters exceeding 0.99. Clinicians managing patients with GBM can choose to become certified in treatment planning with NovoTAL and there are potential clinical benefits in their doing so. Unlike NovoTAL mapping performed by manufacturer, which exclusively utilizes postcontrast MRI sequences, a treating physician has access to a patient’s sequential imaging and has comprehensive knowledge of a patient’s treatment history. This can provide a better understanding of subtle changes in imaging that may or may not represent active disease. In addition, they have the discretion to integrate other MRI or advanced imaging sequences into the planning process, which may better reflect the true extent of active disease (such as T2, FLAIR, diffusion weighted imaging, or MR perfusion), or rely on these sequences at their discretion in instances when postcontrast sequences are missing. In addition, should a patient’s imaging change significantly from baseline while on TTFields, the managing physician can decide whether re-mapping the treatment field is warranted [15].

## Conclusion

Glioblastomas can have a diverse appearance within MRI images. In general, they appear as an enhancing lesion(s) on T1 post-contrast imaging. However, after treatment, not all contrast enhancement represents tumor and may actually depict treatment effect such as pseudoprogression. This needs to be taken into consideration by the treating physician, as shown by several of the cases above. Also, as demonstrated in the cases above, there are often circumstances where the active tumor is non-enhancing, and can only be visualized on FLAIR, MR-perfusion, or diffusion sequences. The lack of contrast-enhancing disease seen on MRI should certainly not preclude patients from receiving TTFields therapy. Future prospective studies may seek to understand not only the response rates of nonenhancing and other MRI sequences of glioblastoma treated with TTFields therapy, but also their correlation with molecular alterations in this tumor.

## Consent

Written informed consent was obtained from the patients and/or caregivers for publication of these Case reports and any accompanying images. A copy of the written consent is available for review by the Editor of this journal.

## Abbreviations

FDA: Food and Drug Administration
FLAIR: Fluid-attenuated inversion recovery
GBM: Glioblastoma
MRI: Magnetic resonance imaging
RANO: Response Assessment in Neuro-Oncology
RT: Radiation therapy
TTFields: Tumor treating fields
